# Computed tomography with a stomach protocol and virtual gastroscopy in the staging of gastric cancer: an initial experience

**DOI:** 10.1590/0100-3984.2017.0097

**Published:** 2018

**Authors:** Maria Fernanda Arruda Almeida, Leonardo Verza, Almir Galvão Vieira Bitencourt, Camila Silva Boaventura, Paula Nicole Vieira Pinto Barbosa, Rubens Chojniak

**Affiliations:** 1 MD, PhD, Radiologist at A.C.Camargo Cancer Center, São Paulo, SP, Brazil; 2 MD, Resident in the Department of Imaging of the A.C.Camargo Cancer Center, São Paulo, SP, Brazil; 3 MD, Radiologist at A.C.Camargo Cancer Center, São Paulo, SP, Brazil; 4 MD, PhD, Coordinator of the Computed Tomography Sector of the A.C.Camargo Cancer Center, São Paulo, SP, Brazil; 5 MD, PhD, Director of the Department of Imaging of the A.C.Camargo Cancer Center, São Paulo, SP, Brazil

**Keywords:** Stomach neoplasms, Multidetector computed tomography, Neoplasm staging

## Abstract

**Objective:**

To evaluate the accuracy of multidetector computed tomography with a stomach protocol in staging of gastric cancer.

**Materials and Methods:**

We evaluated 14 patients who underwent computed tomography in a 16-channel scanner for preoperative staging of gastric adenocarcinoma between September 2015 and December 2016. All images were analyzed by the same radiologist, who had extensive experience in abdominal cancer imaging. The sensitivity, specificity, and accuracy of the method were calculated by comparing it with the pathology result. All patients underwent partial or total gastrectomy.

**Results:**

The mean age was 61.5 years, and 53.8% of the patients were male. The gastric lesions were classified as T1/T2 in 35.7% of the cases, as T3 in 28.5%, and as T4 in 35.7%. Eleven patients (68.7%) had suspicious (N positive) lymph nodes. The accuracy of the T1/T2, T3, T4, and lymph node staging tests was 85%, 78%, 90%, and 78%, respectively. The respective sensitivity and specificity values were 71% and 100% for T1/T2, 66% and 81% for T3, 100% and 90% for T4, and 88% and 60% for lymph nodes.

**Conclusion:**

Multidetector computed tomography with a stomach protocol, used in conjunction with virtual gastroscopy, shows good accuracy in the tumor and lymph node staging of gastric adenocarcinoma.

## INTRODUCTION

Gastric cancer is one of the most prevalent cancers in the world and one of the leading causes of cancer-related death^(1,2)^. Although mortality is decreasing, it is an aggressive tumor, usually detected in advanced stages; and less than 20% of gastric cancer patients survive five years^(1,3,4)^. If the disease is diagnosed early, however, treatment has curative potential, with an average survival of five years in over 90% of cases^(1,3)^.

The main factors related to prognosis and therapeutic planning are tumor depth, lymph node involvement, and distant metastasis^(5,6)^. In Brazil, the tumor-node-metastasis (TNM) system, maintained jointly by the International Union for Cancer Control and the American Joint Committee on Cancer^(7,8)^, currently in its eighth edition, is used for cancer staging, although the classification presented in the seventh edition is maintained, as follows:

- T1 - Tumor invades the lamina propria, muscularis mucosa, or submucosa:
  T1a: tumor invades the lamina propria or muscularis mucosa
  T1b: tumor invades the submucosa
- T2 - Tumor invades the muscularis propria
- T3 - Tumor invades the subserosa without invading the visceral peritoneum
- T4a - Tumor invades the serosa (visceral peritoneum)
- T4b - Tumor invades adjacent structures

Since 1980, endoscopic ultrasound has been used as the gold standard method to evaluate the depth of gastric wall involvement and the local extent (T stage) of gastric tumors^(3,4,9)^. However, it has disadvantages: it is an invasive method, is operator dependent, requires sedation, and is limited to stenotic lesions^(7,9)^. In spite of revealing detail of the lesion, it is not suitable for evaluating distant metastases, lymph node involvement, or peritoneal dissemination^(10,11)^. In addition, its availability is limited in Brazil, even in large population centers, and it is quite costly.

Computed tomography (CT) is routinely used for cancer staging. Specifically for gastric cancer, it is useful for identifying distant metastases and the direct invasion of the tumor into adjacent organs^(1,12)^. However, there have been great technological advances in recent decades. Multidetector CT (MDCT) allows the acquisition of thinner slices and shorter acquisition times, significantly improving image resolution, as well as the use of multiplanar reformatting techniques and three-dimensional reconstruction. The use of these different reconstruction techniques increases the diagnostic precision for the detection of subtle mucosal lesions that can not be detected on two-dimensional images, providing an overall view of the stomach, with the exact location of the tumor, and precise staging, thus contributing to effective treatment planning^(13,14)^. Currently, with high-quality MDCT studies, it is possible to evaluate the extent to which the lesion has invaded the perigastric fat tissue, as well as to detect regional lymph node disease and signs of limited peritoneal carcinomatosis^(6,10,12,15,16)^.

In recent years, studies have demonstrated that with adequate gastric distension, MDCT is capable of detecting small lesions, as well as evaluating the depth of tumor invasion of the gastric wall layers, differentiating T1/T2 lesions from T3 and T4 lesions^(9,10,15,16)^. MDCT with a stomach protocol also allows the use of virtual gastroscopy (VG), a three-dimensional reconstruction technique with intraluminal navigation, specific software being employed to detect alterations in the mucosa and submucosa of the gastric wall^(1,9,10,17)^.

## MATERIALS AND METHODS

This was a retrospective, cross-sectional, single-center study, involving the review of CT images with a stomach protocol and VG for the staging of gastric cancer, with pathological correlation. Prior to the collection of data, the study was approved by the ethics committee of the institution.

The study included 16 patients who underwent MDCT with a stomach protocol, at a referral center for cancer, between September 2015 and December 2016. Of those 16 patients, two were excluded: one for being lost to follow-up; and the other for not undergoing surgery. All of the patients included in the study underwent total or partial gastrectomy, together with lymphadenectomy. In all cases, the surgical specimen was submitted to a standard histopathological study, with evaluation of the depth of the lesion in the layers of the gastric wall, tumor extension into the perigastric peritoneum, and lymph node involvement, as well as TNM classification. The T and N staging by MDCT with a stomach protocol was compared with the final pathological stage, for analysis of the sensitivity, specificity, and accuracy of the technique.

### CT protocol

CT scans were performed in a 16-channel multidetector scanner (Brilliance Big Bore; Philips Healthcare, Cleveland, OH, USA), in an axial plane volumetric acquisition with 1.25 mm collimation. For a better evaluation of the gastric wall, we used a stomach protocol with the following specifications: an 8-h fast; intravenous administration of an antispasmodic agent 10 min prior to the examination; and ingestion of two effervescent salt envelopes, diluted in 10 mL of water, immediately prior to image acquisition.

After images of the upper abdomen had been acquired in the pre-contrast phase, a dynamic study was performed with the following parameters: intravenous injection of nonionic iodinated contrast (Optiray; Mallinckrodt Inc., Raleigh, NC, USA), at a volume of 85-100 mL (depending on patient weight) and a flow velocity of 2.5-3.0 mL/s; images of the upper abdomen acquired in the arterial phase; images of the upper abdomen and pelvis acquired in the portal phase; and images of the upper abdomen and pelvis acquired in the equilibrium phase.

In the equilibrium phase, images should be acquired in the supine position that which ensures the best distension of the stomach portion where the lesion is located (dorsal, right oblique, or left oblique).

### T staging and image analysis

All images were analyzed by the same radiologist, who had extensive experience in abdominal cancer imaging. The radiologist was aware that the images corresponded to the examinations of patients diagnosed with gastric cancer, as well as having access to information regarding the size and location of the lesion at endoscopy. However, the radiologist was blinded to the pathology findings.

The T staging was performed through analysis of axial images, as well as and coronal and sagittal reformatted images, followed by intraluminal navigation through threedimensional reconstruction using specific software- Aquarius iNtuition program (TeraRecon, Inc., Foster City, CA, USA) or Vitrea Workstation (Canon Medical Systems do Brasil, Campinas, Brazil). Imaging criteria for evaluation of the extent of lesions in the gastric wall were the same as those used in previous MDCT studies^(1,5,6,9,17)^, consistent with the TNM classification^(7,8)^ and characterized as follows:

- T1 - No changes seen in the gastric wall-There is focal thickening of the wall, with or without enhancement of the inner layer, and a low density band at the base of the lesion corresponding to the preserved submucosal layer; that is, focal enhancement only, without wall thickening.
- T2 - Complete thickening of the gastric wall, accompanied by disappearance or interruption of the low density range, the external contour remaining well-defined and smooth-There is no bulging of the external gastric contour by the tumor, and the perigastric fat tissue is preserved.
- T3 - Complete thickening of the gastric wall, accompanied by disappearance or interruption of the low density range, the external contour remaining well-defined, albeit with slight bulging of the outer layer caused by the tumor-The perigastric fat tissue is preserved.
- T4 - Nodular or irregular thickening of the outer layer of the gastric wall surrounding the tumor-There is increased density of the perigastric fat tissue or invasion of adjacent structures/organs.

## RESULTS

During the study period, 19 patients underwent MDCT with a stomach protocol. Of those 19 patients, three had no histological diagnosis of adenocarcinoma by endoscopic biopsy (the diagnosis being gastrointestinal stromal tumor in two and leiomyoma in one) and were excluded from the analysis. Of the remaining 16 patients, one was lost to follow-up and was therefore also excluded. Another patient was excluded due to M1 clinical staging, based on a finding of peritoneal carcinomatosis on tomography, and therefore received only systemic treatment with chemotherapy.

The final sample comprised 14 patients. The mean age was 61.5 years (range, 31-89 years), and 53.8% were male. All patients had histological confirmation of adenocarcinoma, and the majority (55.2%) had poorly or moderately differentiated adenocarcinoma. In all cases, the MDCT images acquired with a stomach protocol and VG made it possible to identify the gastric tumor and were considered appropriate for staging.

The gastric lesions were classified as T1/T2 in 35.7% of the cases, as T3 in 28.5%, and as T4 in 35.7%. In terms of the lymph node staging, 11 patients (corresponding to 68.7% of the cases) were classified as N-positive (with suspicious lymph nodes). In 75% of the cases, abdominal CT did not show distant metastases (M0). Thirteen patients underwent surgical resection, and eight (61.5%) of those patients received neoadjuvant chemotherapy. The pathological staging showed the following: signs of invasion of the submucosa or lamina propria mucosae (T1) in five patients (35.7%); signs of invasion of the muscle layer (T2) in two (14.2%); invasion of the serosa (T3) in three (21.4%); and signs of invasion of the peritoneum or neighboring structures (T4) in three (21.4%). A patient with T4N+ staging by CT had a proposed surgical resection canceled after the identification of a T4 lesion (perigastric peritoneal infiltration), together with peritoneal carcinomatosis in a staging laparoscopy, as confirmed through biopsy. That patient was therefore classified as cT4yN3pM1.

The sensitivity and specificity were calculated separately for three groups-T1/T2, T3, and T4-as shown in Table 1, depending on the level of agreement between the CT staging and the pathology (Table 2).

**Table 1:** Sensitivity, specificity and accuracy of MDCT for T and N staging.

Staging	Sensitivity	Specificity	Accuracy
T1/T2	71%	100%	85%
T3	66%	81%	78%
T4	100%	90%	90%
Lymph nodes	88%	60%	78%

**Table 2:** Comparison between MDCT staging and pathological staging.

	MDCT	Pathological	Agreement	Agreement
Patient	staging	staging	T staging	N staging
1	T1/T2, N0	T1b, N1	Yes	FN
2	T4a, N+	T4a, N1	Yes	TP
3	T1/T2, N+	T1b, N0	Yes	FP
4	T3, N+	T1b, N0	No	FP
5	T4a, N+	T4a, N3	Yes	TP
6	T3, N+	T2, N3b	No	TP
7	T3, N+	T3, N3b	Yes	TP
8	T1/T2, N0	T2, N0	Yes	TN
9	T1/T2, N0	T1a, N0	Yes	TN
10	T3, N+	T3, N1	Yes	TP
11	T4a, N+	T4a, N2	Yes	TP
12	T1/T2, N0	T1a, N0	Yes	TN
13	T4a, N+	T4a, N1	Yes	TP
14	T4a, N+	T3, N3a	No	TP

FN, false-negative; TP, true-positive; FP, false-positive; TN, true-negative.

## DISCUSSION

With advances in therapeutic modalities and greater evidence of the benefit of neoadjuvant chemotherapy for the T3, T4, and N+ stages of gastric cancer, imaging techniques now contribute significantly to the therapeutic decision-making process^(18)^.

The gold standard technique for local staging of gastric cancer is endoscopic ultrasound. In a systematic meta-analysis, Kwee et al. demonstrated that endoscopic ultrasound has variable rates of accuracy in the diagnosis of T staging (65-92%), as well as a sensitivity of 70.8% and a specificity of 84.6% for the detection of perigastric lymph node involvement^(19)^.

**Figure 1:**
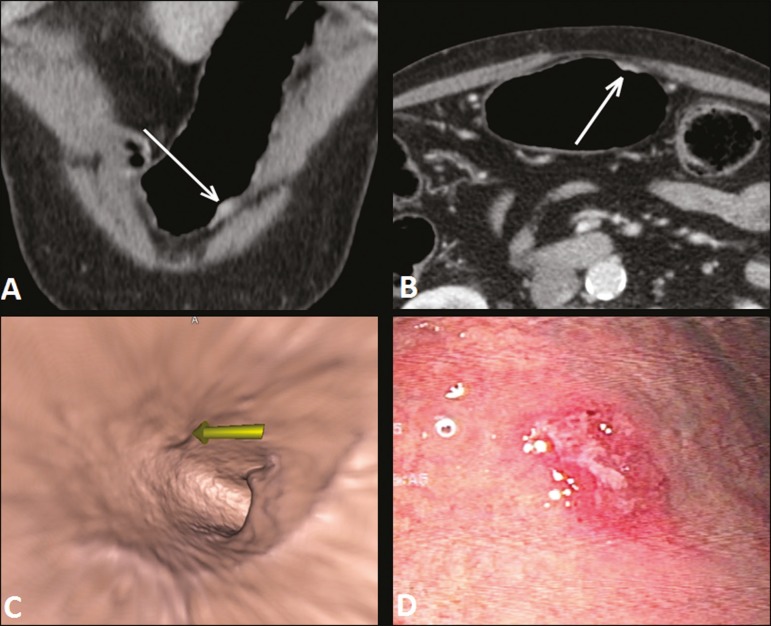
**A,B:** Coronal and axial MDCT scans showing focal thickening (arrows) of the anterior wall of the great curvature of the gastric body, with post-contrast enhancement sparing the serosa. **C:** Three-dimensional reconstruction with VG study showing an irregular lesion on the inner surface of the stomach, suggestive of ulceration. **D:** Gastrointestinal endoscopy demonstrating an infiltrative lesion with hyperemia in the distalbody of the great curvature. MDCT staging: T1/T2N0; pathological staging: T1N0.

Initial studies comparing local staging by endoscopic ultrasound and CT have produced disappointing results^(20)^. However, in recent years, there have been great technological advances in MDCT, allowing the acquisition of high-resolution images with fine slices, as well as multiplanar and three-dimensional reconstructions. All of those improvements, combined with the development of new protocols, have contributed to an increase in CT sensitivity and accuracy in the local staging of gastric cancer^(9)^. In addition, software for intraluminal navigation has facilitated the detection of early lesions of the mucosa and submucosa (T1/T2 lesions), as shown in Figure 1, because it provides an evaluation of the internal surface of the organ. The virtual images generally exceed the temporal limits of optical endoscopy of the stomach in the evaluation of lesions of the small curvature and duodenal bulb, as well as allowing retrospective navigation to evaluate possible blind spots^(21)^. CT also has the advantages of being noninvasive, widely available, and relatively inexpensive.

Application of a stomach protocol guarantees optimum gastric distension, being fundamental for the adequate evaluation of focal thickening of the gastric wall and visualization of the internal surface of the stomach through VG. A collapsed stomach can obscure an injury or mimic an illness.

Recent studies have demonstrated that MDCT with a stomach protocol has an accuracy similar to that of endoscopic ultrasound^(9,22)^. Kim et al.^(12)^ reported that MDCT with a stomach protocol has a sensitivity of 62-93% and a specificity of 90-97%. Another study evaluating the accuracy of MDCT in the T staging of gastric cancer, using axial analysis (two-dimensional mode) alone or in combination with multiplanar analysis and three-dimensional reconstruction^(13)^, also showed significant improvement in diagnostic performance (73% vs. 89% for the combination), reaffirming the importance of combining these techniques in the evaluation of images.

Our work did not focus on differentiating between T1 and T2 lesions, although some authors have argued that MDCT with a stomach protocol can achieve the image resolution necessary for such a distinction. From a clinical point of view, the differentiation between T1 and T2 lesions is not fundamental, given that neoadjuvant therapy is not recommended in either stage. We designated the groups T1/T2, T3, and T4, using the new criteria for patterns of gastric wall involvement. The values of sensitivity, specificity, and accuracy were similar to those reported in other studies. In three patients, there was disagreement between the MDCT staging and the pathological staging. Two of those patients were staged as T3 by MDCT, whereas they were staged as T1b and T2, respectively, in the final pathological staging. The third patient was staged as T4 by MDCT and as T3 in the final pathologic staging. It is noteworthy that all three of those patients were submitted to neoadjuvant chemotherapy after the MDCT staging, and the disagreement between the two staging methods might result in downstaging due to the therapeutic response. There were no cases of lesions for which the pathological staging was higher than that of the imaging. In the remaining patients, there was agreement between the MDCT staging and the pathological staging.

Some borderline T2/T3 lesions can be difficult to define in MDCT. There are factors that can hamper differentiation, such as the variation in the amount of fat tissue from patient to patient and a reduced area of enhancement if there is exaggerated distension of the gastric chamber^(23)^. For borderline lesions, there can still be difficulty in quantifying the blurring of the perigastric plane. Some T2 lesions may cause discrete blurring of the perigastric fat by a desmoplastic reaction. Habermann et al.^(22)^ used the same criterion employed in endoscopic ultrasound to differentiate between desmoplastic reaction and tumor infiltration; that is, when the blurring affects less than onethird of the tumor border, the lesion is classified as T2, and when it is more extensive, the lesion is classified as T3. The authors found that there was no incorrect staging of T2 tumors when that criterion was applied.

**Figure 2:**
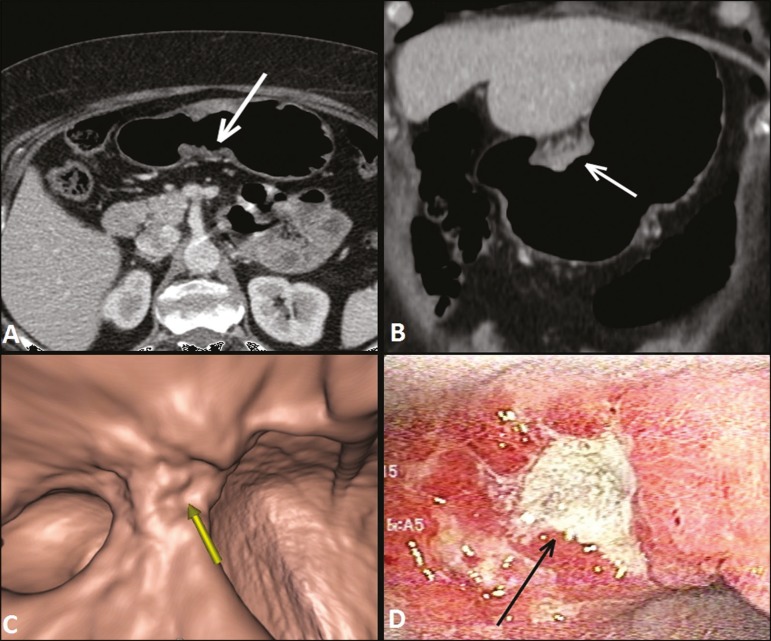
**A,B,C:** Axial MDCT scan, coronal MDCT scan, and VG study (yellow arrow), showing an ulcerated lesion at the incisura angularis (arrows). **D:** Endoscopic image showing an ulcerated, friable lesion at the same location (arrow). MDCT staging: T3; pathological staging: T3.

**Figure 3:**
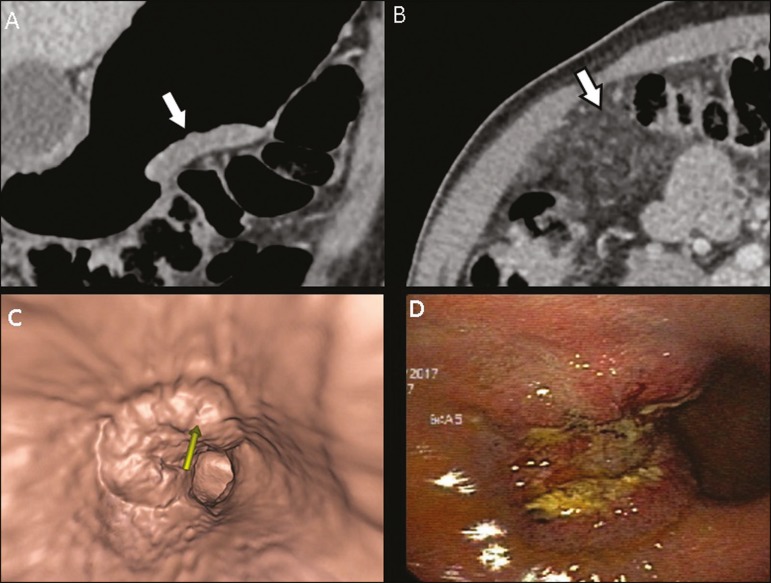
**A,B:** Coronal and axial MDCT scans showing irregular parietal thickening with post-contrast enhancement affecting the great curvature (arrow), associated with fat blurring in the right hypochondrium (arrow), suggestive of peritoneal carcinomatosis. **C,D:** VG and endoscopy, showing an ulcerated, elevated lesion. MDCT staging: T4a; pathological staging: T4a.

**Figure 4:**
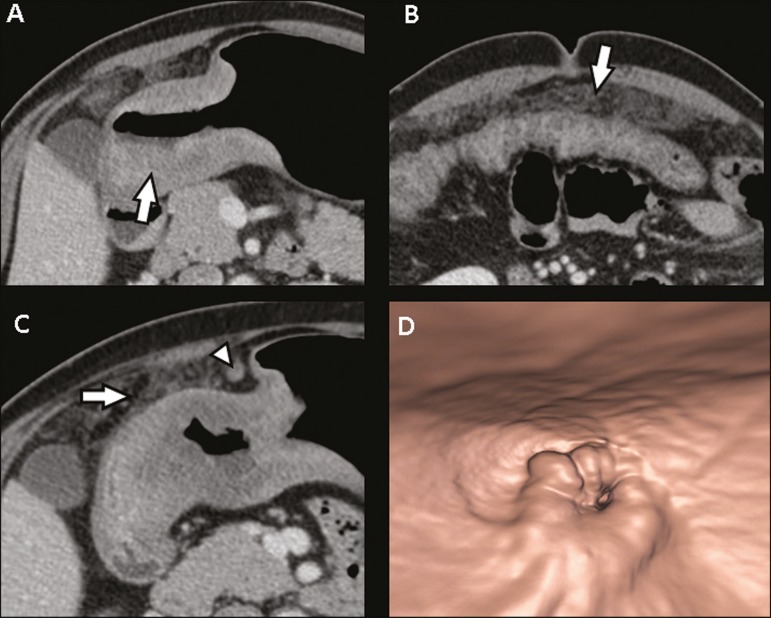
**A,B:** MDCT axial section showing thickening and enhancement of the entire wall (arrow), accompanied by nodular densification of the peritoneum, suggestive of peritoneal carcinomatosis (arrow). **C:** Obliteration of perigastric fat planes (arrow) and enlargement of the adjacent lymph nodes (arrowhead). **D:** VG study showing stenosis in the antropyloric region. MDCT staging: T4N+; pathological staging: T4bN2.

The differentiation between T3 (Figure 2) and T4 lesions (Figures 3 and 4) is very important, given the surgical difficulty of deep lesions or those extending to neighboring structures. The differentiation is also important in the indication of associated therapies such as intraperitoneal hyperthermic chemotherapy. T4a tumors generally present more evident blurring of perigastric fat than do T3 tumors, with dense striation or even a nodular appearance^(5,12)^. For T4b lesions, there is direct invasion of adjacent structures or organs. Three-dimensional reconstruction is useful and should be used in order to differentiate between these forms of involvement.

Lymph node involvement is one of the factors that directly influences patient prognosis. In a meta-analysis, Kwee et al.^(11)^found that, for lymph node staging, MDCT has a sensitivity of 62.5-93.0% and a specificity of 90.5-97.9%. One of the reasons for the differences across studies is the variety of criteria used in order to classify a lymph node as affected, the most widely used parameter being the size, with a cut-off threshold ranging from 5 to 10 mm at its smallest diameter^(23)^. We defined affected perigastric lymph nodes as those measuring > 6 mm at their smallest diameter or ≥ 8 mm at their largest diameter. Another valued criterion is the presence of necrosis^(14)^. In our study, the sensitivity and specificity values were 60% and 80%, respectively.

It should be borne in mind that VG does not allow the evaluation of perigastric tumor involvement, diagnosis of regional lymph node diseases, or identification of distant metastases; VG findings should always be interpreted together with the analysis of axial plane images and multiplanar reconstructions^(13)^. Other limitations of VG are related to the specific examination protocol, which requires preparation of the patient with prolonged fasting (for 8 h), the use of additional medications, and the presence of a radiologist. It also requires additional radiologist time to perform three-dimensional reconstruction and intraluminal navigation, adding an average of 20-30 min per patient.

Our study has certain limitations. The small number of patients and the fact that some underwent neoadjuvant chemotherapy increased the likelihood of disagreement among the findings. Despite our initial experience, we believe that MDCT with a stomach protocol brings benefits in the clinical staging of gastric cancer, as demonstrated in several international studies. Further studies with larger patient samples may help confirm the results that have been presented in recent years, as well as allowing the impact of CT on the management of these patients to be evaluated.

In conclusion, MDCT has become an accessible, noninvasive method for the evaluation of the local extent and lymph node involvement in gastric cancer staging. When performed with a stomach protocol, MDCT provides additional information to improve the therapeutic decisionmaking process, showing good accuracy when differentiating among T1/T2, T3, and T4 lesions, and should be considered a viable alternative to endoscopic ultrasound.
